# Barriers and facilitators to structured, out-of-school physical activity participation among rural youth in the United States: a systematic review

**DOI:** 10.1186/s12889-026-26205-x

**Published:** 2026-01-31

**Authors:** Ashleigh M. Johnson, Tyler Prochnow, Zachary Townsend, Carissa R. Smock, Cassandra M. Beattie, Lora Peterson, M. Renée Umstattd Meyer, Christopher D. Pfledderer

**Affiliations:** 1 School of Exercise and Nutritional Sciences, San Diego State University, 5500 Campanile Drive, San Diego, CA 92182 USA; 2 School of Public Health, Texas A&M University, 400 Bizzell St., College Station, TX 77843 USA; 3 School of Health Sciences, Salisbury University, 1101 Camden Avenue, Salisbury, MD 21801-6860 USA; 4 Department of Leadership, Management, and Human Capital, School of Business and Economics, National University, 9388 Lightwave Ave, San Diego, CA 92123 USA; 5 Institute for Advancing Health Through Agriculture, Texas A&M Agrilife, 578 John Kimbrough Blvd., College Station, TX 77843-2147 USA; 6 National University, 9388 Lightwave Ave, San Diego, CA 92123 USA; 7 Department of Public Health, Robbins College of Health and Humans Sciences, Baylor University, 1311 S 5th St, Waco, TX 76706 USA; 8 School of Public Health in Austin, The University of Texas Health Science Center Houston, 1836 San Jacinto Blvd., Suite 510, Austin, TX 78701 USA

**Keywords:** Geographic disparities, Youth, Ecological model, Systematic review, Physical activity

## Abstract

**Background:**

Barriers to physical activity (PA) participation affect United States (US) youth’s ability to meet PA guidelines. There is evidence showing lower PA among rural versus urban youth due to fewer PA opportunities and resources. Out-of-school programs can help reduce geographic disparities in PA, but there is a dearth of literature on participation factors among rural youth. This study aimed to identify, describe, and synthesize peer-review literature on barriers and facilitators to participation in structured, out-of-school PA at multiple ecological levels for rural-dwelling, US youth.

**Methods:**

A systematic review was conducted November 2024 using Medline, PubMed, SPORTDISC, Web of Science, APA Psychinfo, and CINAHL for articles published 2000–2024. Articles needed to be (1) peer-reviewed; (2) English-language; (3) conducted among US rural populations; (4) examining barriers and/or facilitators to out-of-school, structured PA; and (5) conducted among youth ages 6–17. Articles focusing on participants with additional needs were excluded.

**Results:**

A search of 3,070 articles was refined to a final sample of 39 articles. Participant factors were identified at multiple ecological levels, including environmental and program (programs and facilities, transportation and accessibility, resources and infrastructure, safety, cost/fees); social (family support and role models, peer support, lack of social support); and individual (interest, motivation, and enjoyment; skill development; time constraints and prioritization) levels.

**Conclusions:**

Rural youth participation in structured, out-of-school PA is informed by multi-level factors, including facilities and programs, transportation, resources and infrastructure, social support, and interest/motivation. Additional research is needed to examine participation factors among this population. Findings can be used to design and adapt out-of-school programs that meet the unique needs of youth in rural settings.

**Supplementary Information:**

The online version contains supplementary material available at 10.1186/s12889-026-26205-x.

## Background

Only about 20% of American youth meet United States (US) Department of Health and Human Services aerobic physical activity (PA) guidelines[1], defined as at least 60 min of moderate- to vigorous-intensity physical activity (MVPA) per day [2]. Among youth, regular PA is associated with improved weight status, cardiorespiratory and muscular fitness, cognition, and bone health [3]. Low levels of activity among children can also have long-term implications, as they contribute to obesity in young adulthood and chronic disease development, including unfavorable cardiometabolic risk profile and impaired glucose metabolism in adulthood [4–7]. 

Despite the numerous health benefits of PA, there is evidence of geographic disparities in organized PA opportunities for youth. Rural youth, which account for about 20% of school-age youth in the US[8], face unique challenges to PA participation (e.g., organized sports and free play) compared to their urban and suburban counterparts. This includes fewer opportunities and resources for PA, such as fewer recreational centers and organized sports leagues [9]. These challenges are often compounded by geographic isolation, long travel distances to available facilities, and socioeconomic factors that make it difficult for rural youth to be active within their community [9]. 

In line with the Comprehensive School Physical Activity Program (CSPAP)[10], out-of-school programs play an important role in supporting regular PA among youth. The goals of these comprehensive programs are diverse, as these opportunities typically include a variety of activities that can keep youth active (e.g., socializing activities, academic enrichment, arts and crafts, sports, play) [11]. They provide a structured, supervised environment for PA outside of regular school hours, and have been shown to contribute to daily MVPA [12, 13]. Such programs, including the YMCA, Boys & Girls Clubs, and 4-H, can provide consistent opportunities for PA while supporting additional youth needs, such as skill development, teamwork, and social interaction [14]. Out-of-school PA programs have the potential to reduce geographic disparities in access to PA opportunities, particularly in underserved areas where resources may be limited. These programs provide a centralized, often school- or community center-based location for youth activity, which helps to overcome challenges of long travel distances [15]. Data from a 2016 report by the Afterschool Alliance show an estimated 18% of youth nationwide attend an out-of-school program, compared to 13% of youth in rural communities, although nearly 40% express an interest in participating [16, 17]. To effectively address existing needs and boost rural youth participation in these programs, a comprehensive understanding of the factors that influence engagement is crucial for program development and improvement.

To comprehensively understand the complex factors influencing rural youth participation in out-of-school PA programs, a social ecological model approach provides a valuable theoretical framework. The social ecological model recognizes that health behaviors, including PA, are influenced by multiple, interconnected levels of factors rather than individual characteristics alone [18]. At the individual level, factors such as personal motivation, self-efficacy, physical competence, previous activity experiences, and demographic characteristics may influence a rural youth’s likelihood to participate in structured PA programs. The social level encompasses interpersonal relationships and social networks, including family support and attitudes toward PA, peer influences, social norms within the rural community, and the role of mentors or program staff in encouraging participation. Finally, the environment and program level addresses organizational and community factors that may facilitate or hinder participation, such as program accessibility and transportation options, facility availability and quality, program costs, scheduling compatibility with family obligations, and broader community culture surrounding youth PA. This multilevel approach allows for a more nuanced understanding of the interconnected barriers and facilitators that rural youth face when considering participation in out-of-school PA programs.

Understanding participation factors can promote enrollment and inform the design of effective, sustainable, and equitable out-of-school programs tailored to the unique needs of rural communities. By applying a social ecological lens to examine these participation factors, researchers and practitioners can identify intervention points across multiple levels and develop more comprehensive, contextually appropriate strategies for increasing rural youth engagement. Currently, existing reviews of the literature are focused on barriers and facilitators of PA more broadly[15, 18], while reviews examining participation in out-of-school or organized PA programs are focused on special populations (e.g., psychiatric or autism spectrum disorders), limiting generalizability to rural youth [19, 20]. Despite a growing body of research on youth PA and rural health disparities, a comprehensive synthesis of the literature remains a critical gap. This systematic review will organize findings within the social ecological framework to provide actionable insights for developing more effective out-of-school PA programs for rural youth populations. Thus, the purpose of this study was to identify, describe, and synthesize peer-review literature on barriers and facilitators to participation in structured, out-of-school PA at multiple ecological levels for US youth living in rural areas.

## Methods

### Literature search

This systematic review was conducted and is reported according to PRISMA guidelines [21]. A search of the literature was conducted in November 2024 using Medline, PubMed, SPORTDISC, Web of Science, APA Psychinfo, and CINAHL databases. Search terms used for this search are provided in Supplementary File 1. All records were imported into Covidence to facilitate the review process [22]. 

### Inclusion and exclusion criteria

To be included in the final sample, articles needed to (1) peer-reviewed; (2) English-language; (3) conducted among US rural populations; (4) examining barriers and/or facilitators to out-of-school, structured PA; and (5) conducted among youth ages 6–17. Studies were limited to those conducted in the US to ensure contextual relevance and applicability of findings to US-based rural communities, where educational systems, recreation infrastructure, and policy frameworks may differ substantially from other countries. No strict definition of rural was used, as long as the article in question self-reported the study population or community as rural. Our search was limited to articles published between 2000 and 2024. Articles were excluded if they focused on participants with additional needs (e.g., youth with asthma).

### Data extraction and quality assessment

Eight independent reviewers (AJ, TP, CS, ZT, CB, MRUM, LP, CP) reviewed titles/abstracts against the inclusion and exclusion criteria and conducted full text review. The reviewers used a data extraction template to gather descriptive information from the final sample (Supplementary File 2), including: (1) study type, (2) setting, (3) location, (4) rural definition, (5) participant type, (6) program participation factors (i.e., barriers and facilitators), and (7) data collection approach. Each article was extracted by one of the reviewers and data checked by another reviewer. Program participation factors were analyzed through an ecological lens (i.e., classified as an individual-, social-, or program/environmental-level factor).

The Appraisal tool for Cross-Sectional Studies (AXIS) was used to systematically assess the studies and examine reliability of the data presented [23]. This 20-item checklist is designed to inform decisions about the quality of the study being appraised and includes items in three main categories: quality of study design (7 items), reporting quality (7 items), and introduction of biases (6 items). The authors assessed study quality on a scale of “yes” (1 point), “no” (0 points) or “don’t know” (0.5 points) for 20 possible points (items 13 and 19 were reverse coded). AXIS does not provide an established rule for determining the quality of each study. Previous quality assessments using AXIS have used predetermined percentage values to classify publications as high, moderate, or lowquality [24–27]. For the present study, authors used the predetermined values of ≥ 70% equals high quality/low risk of bias; 50–69% equals moderate quality/moderate risk of bias; and < 50% equals low quality/high risk of bias.

## Results

**Fig. 1:**
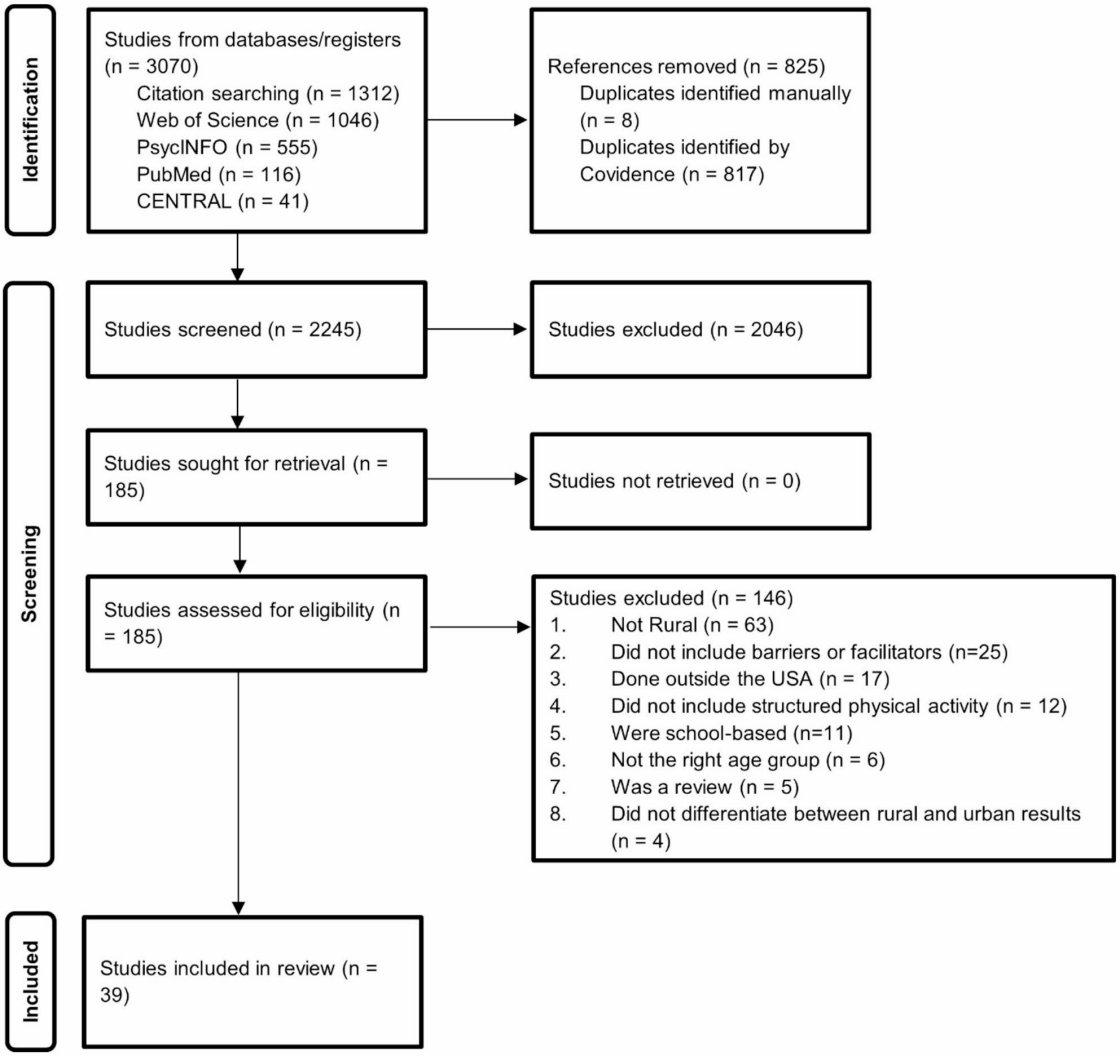
PRISMA Diagram

The initial search resulted in 3,070 articles. Duplicates (*n* = 825) were removed prior to abstract review, resulting in 2,245 for title and abstract screening. After title and abstracts were reviewed against the inclusion and exclusion criteria, 2,060 articles were removed. The remaining articles (*n* = 185) were assessed by two reviewers independently. Lead and senior authors (AJ, CP) discussed any conflicts until agreement was reached. In total, 146 articles were excluded during the full-text review because they were (1) not a rural population (*n* = 63), (2) did not include barriers or facilitators (*n* = 25), (3) were conducted outside of the US (*n* = 17), (4) did not include structured PA participation (*n* = 12), (5) were school-based (*n* = 11), (6) were not conducted among youth ages 6–17 (*n* = 6), (7) was a review article (*n* = 5), (8) did not differentiate between rural and urban results (*n* = 4), or (9) were not peer reviewed (*n* = 3). The final sample contained 39 articles, which were then moved to data extraction. Figure 1 presents the Preferred Reporting Items for Systematic Reviews and Meta-Analyses (PRISMA) data and diagram.

**Fig. 2:**
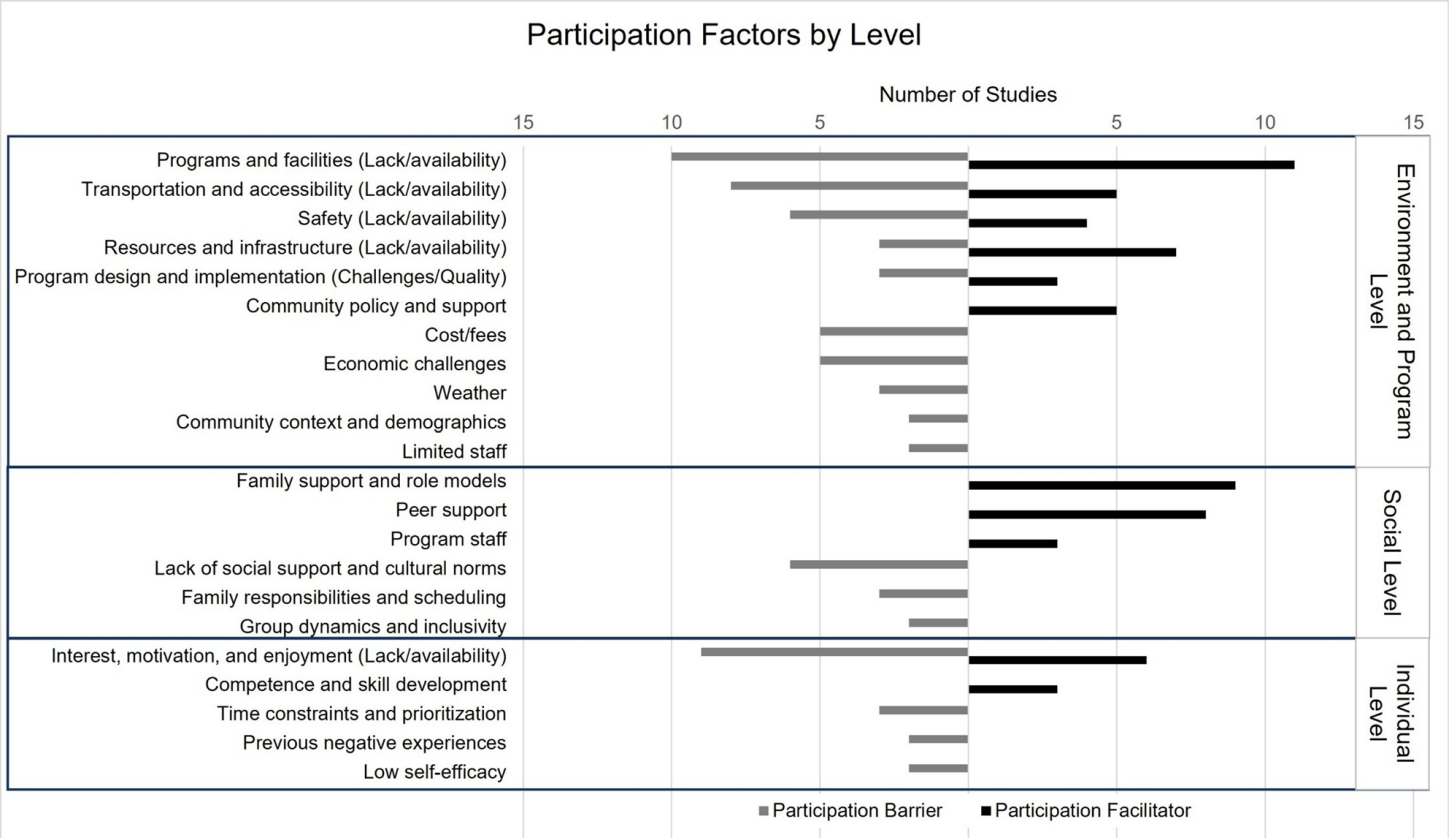
Participation factors of structured, out-of-school physical activity by ecological level

A majority of the articles (*n* = 24; 61.5%) were published between 2015 and 2024. Roughly half of articles were missing a definition of rural (*n* = 19; 48.7%), followed by use of a generic urban/rural classification (*n* = 8; 20.5%), Rural Urban Commuting Area (RUCA) system (*n* = 6; 15.4%), NCES (National Center for Educational Statistics) classification system (*n* = 3; 7.7%), United States Department of Education (*n* = 1; 2.6%), over 10 miles from an urbanized area (*n* = 1; 2.6%), and Utah State Office of Education (*n* = 1; 2.6%) (Table 1). Of the 39 studies extracted in this study, 74.4% (*n* = 29) included youth perspectives on barriers and facilitators of participation in structured, out-of-school PA opportunities, 35.9% (*n* = 14) included parent perspectives, 25.6% (*n* = 10) included staff perspectives, and 12.8% (*n* = 5) included perspectives from the broader community (Table 2). Figure 2 presents the participation factors of structured, out-of-school PA by ecological level.

**Table 1:** Study information from review sample

Citation	Title	Location	Participant Type (*n*)	Participant Characteristics	Rural Definition
Alexander et al., 2015 [28]	Childhood Obesity Perceptions Among African American Caregivers in a Rural Georgia Community: A Mixed Methods Approach	Georgia	Parents - survey (*n* = 135) Parents - interview (*n* = 12)	92–96% Female; 100% Black or African American	Missing
Balvanz et al., 2016 [29]	From Voice to Choice: African American Youth Examine Childhood Obesity in Rural North Carolina	North Carolina	Youth (*n* = 7)	100% Female 100% African American	Missing
Barfield et al., 2021 [30]	Get Outside! Promoting Adolescent Health through Outdoor After-School Activity	Oregon	Youth (*n* = 26) Parents (*n* = 6)	50% Female; 75% White (Youth)	Missing
Brett et al., 2002 [31]	Using Ethnography to Improve Intervention Design	Colorado	Parents (*n* = 29)	62% Hispanic	Generic urban/rural classification
Brown et al., 2018 [32]	Feasibility and outcomes of an out-of-school and home-based obesity prevention pilot study for rural children on an American Indian reservation	Missing	Youth (*n* = 23) Parents (*n* = 23) Staff (*n* = 5)	52% American Indian (Youth); 74% Female (Parents)	Generic urban/rural classification
Chadwick et al., 2019 [33]	Collaborative implementation of a community-based exercise intervention with a partnering rural American Indian community	Oklahoma	Youth (*n* = 90)	100% American Indian	Missing
Christiana et al., 2014 [34]	“I’d Rather Dance Outside”: A Phenomenological Examination of Youth Experiences in Outdoor, Noncompetitive Physical Activity	Southeastern United States	Youth (*n* = 24)	50% Female; 38% Black or African American	Missing
Christiana et al., 2017 [35]	The Role of Competition in Leisure-Time Physical Activity Among Middle School Youth: Implications for Park and Recreation Professionals	Southeastern United States	Youth (*n* = 838)	53% Female; 52% White	Generic urban/rural classification
Edwards et al., 2011 [36]	Opportunities for Extracurricular Physical Activity in North Carolina Middle Schools	North Carolina	Staff (*n* = 325)	Not provided	Modified version of the NCES (National Center for Educational Statistics) classification system
Edwards et al., 2013 [37]	Place Disparities in Supportive Environments for Extracurricular Physical Activity in North Carolina Middle Schools	North Carolina	Staff (*n* = 325)	Not provided	Modified version of the NCES (National Center for Educational Statistics) classification system
Edwards et al., 2014 [38]	Promoting Youth Physical Activity in Rural Southern Communities: Practitioner Perceptions of Environmental Opportunities and Barriers	North Carolina	Staff, Community (*n* = 30)	63–64% Male; 57–100% White	Generic urban/rural classification
Flett et al., 2010 [39]	Connecting Children and Family with Nature-Based Physical Activity	Michigan	Parents (*n* = 19) Youth (*n* = 23)	95% Female	Generic urban/rural classification
Galaviz et al., 2016 [40]	Parental Perception of Neighborhood Safety and Children’s Physical Activity	National	Youth (*n* = 9,827)	51% Female; 57% White	Missing
Gay et al., 2011 [41]	Environmental Determinants of Children’s Physical Activity in Residential Children’s Homes	North Carolina, South Carolina	Youth (*n* = 196) Staff (*n* = 23)	58% Male; 48% White	Rural Urban Commuting Area (RUCA) system
Hennessy et al., 2010 [42]	Active Living for Rural Children Community Perspectives Using PhotoVOICE	California, Mississippi, South Carolina, Kentucky	Parents (*n* = 99) Staff (*n* = 17)	Not provided	NCES (National Center for Educational Statistics) classification system
Hinkle et al., 2018 [43]	How Food & Fitness Community Partnerships Successfully Engaged Youth	Missing	Youth (*n* = 73)	50% Male; 33% Black or African American	Missing
Kasehagen et al., 2012 [44]	Associations Between Neighborhood Characteristics and Physical Activity Among Youth Within Rural–Urban Commuting Areas in the US	National	Youth (*n* = 45,392)	56% Male; 53% White	Rural Urban Commuting Area (RUCA) system
Kellstedt et al., 2021 [45]	Rural community systems: Youth physical activity promotion through community collaboration	Missing	Youth (*n* = 418) Community (*n* = 49)	92–96% White	Other: Distance from an urbanized area (> 10 miles)
Kellstedt et al., 2022 [46]	The COVID-19 pandemic and changes in children’s physical activity in a rural US community: a mixed methods study	Nebraska	Youth (*n* = 318) Community (*n* = 23)	52–57% Female	United States Department of Education
Kristjansson et al., 2015 [47]	Needs assessment of school and community physical activity opportunities in rural West Virginia: the McDowell CHOICES planning effort	West Virginia	Community (*n* = 80) Youth (*n* = 465)	52% Male (Youth)	Generic urban/rural classification
Leslie et al., 2016 [48]	Parent perceptions of a child physical activity initiative in a rural community	Missing	Parents (*n* = 21)	95% Female; 95% White	Missing
MacDowell et al., 2011 [49]	Illinois 4-H Health Jam for Healthy Lifestyles and Rural Pipeline Awareness	Illinois	Youth (*n* = 262)	Not provided	Rural Urban Commuting Area (RUCA) system
McBride et al., 2017 [50]	Motivational Regulations Amongst At Risk Students in an After School Activity Program	Texas	Youth (*n* = 171)	53% Female; 85% Black or African American	Missing
Melton et al., 2018 [51]	Motivation of Rural Parents for Youth Recreational Sports Programs	Georgia	Parents (*n* = 466)	71% Male; 67% White	Missing
Meyer et al., 2019 [52]	Come together, play, be active: Physical activity engagement of school-age children at Play Streets in four diverse rural communities in the U.S.	Maryland, North Carolina, Oklahoma, Texas	Youth (*n* = 376) Parents (*n* = 65)	55% Female, 59% White (Youth); 83% (Adults)	Rural Urban Commuting Area (RUCA) system
Meyer et al., 2021 [53]	The Effects of Play Streets on Social and Community Connectedness in Rural Communities	Maryland, North Carolina, Oklahoma, Texas	Staff (*n* = 14) Parents (*n* = 7) Youth (*n* = 25)	Not provided	Rural Urban Commuting Area (RUCA) system
Molitor & Naber, 2024 [54]	Exploring the feasibility of an occupational therapy afterschool program among rural elementary children	Midwest	Youth (*n* = 23)	65% Female	Missing
Nanney et al., 2008 [55]	Poverty-Related Factors Associated with Obesity Prevention Policies in Utah Secondary Schools	Utah	Staff (*n* = 209)	Not provided	Other: Utah State Office of Education
Palmer et al., 2024 [56]	Perspectives of Rural High School Students Involved in a Multi-Component, After-School Physical Activity Intervention	Midwest	Youth (*n* = 10)	70% Male; 90% White	Missing
Pate et al., 2003 [57]	Evaluation of a Community-based Intervention to Promote Physical Activity in Youth: Lessons From Active Winner	South Carolina	Youth (*n* = 436)	51% Female; 59–87%	Missing
Prochnow et al., 2020 [58]	Differences in Child Physical Activity Levels at Rural Play Streets Due to Activity Type and Sex	Missing	Youth (*n* = 1,750)	58% Female	Rural Urban Commuting Area (RUCA) system
Roberts 2023 [59]	Improving Elementary Students Social-Emotional Health through an After-School Mentorship Physical Activity Program	Pennsylvania	Parents (*n* = 8) Staff (*n* = 20) Youth (*n* = 7)	58–88% Female	Generic urban/rural classification
Shah et al., 2019 [60]	Social Support for Physical Activity for High Schoolers in Rural Southern Appalachia	Appalachia	Parents (*n* = 18) Staff (*n* = 38) Youth (*n* = 21)	72–76% Female	Missing
Sharaievska et al., 2019 [61]	Use of Physical Activity Monitoring Devices by Families in Rural Communities: Qualitative Approach	Appalachia	Parents (*n* = 11)	100% White	Missing
Smith et al., 2022 [62]	Evaluating the effectiveness of ‘mentoring to be active’ for rural Appalachian middle school youth on physical activity and dietary sugar consumption during ‘out of school’ time	Appalachia	Youth (*n* = 52)	52% Female; 87% White	Missing
Thunfors et al., 2009 [63]	Health behavior interests of adolescents with unhealthy diet and exercise: implications for weight management	Pennsylvania	Youth (*n* = 737)	53% Female	Missing
Tosa et al., 2018 [64]	RezRIDERS: A Tribally-Driven, Extreme Sport Intervention & Outcomes	New Mexico	Youth (*n* = 55) Parents (*n* = 5)	52–60% Female; 100% Native American	Missing
Yousefian et al., 2009 [65]	Active Living for Rural Youth: Addressing Physical Inactivity in Rural Communities	Maine	Youth (*n* = 84) Community (*n* = 15)	55% Female	Generic urban/rural classification
Zuest 2020 [66]	Physical Activity Experiences of Adolescent Girls Living in a Rural Community	Sierra Nevada Mountains	Youth (*n* = 11)	100% Female; 73% White	Missing

**Table 2:** Barriers and facilitators of participation in structured, out-of-school physical activity

Environment and Program Level			
Participation Factor	Description	Direction of Impact	Number of Studies (Citations)
Lack of facilities and programs	Limited number of venues, health programs, indoor/outdoor facilities, extracurricular activities; lack of sports available for girls	Barrier	*n* = 10 (Alexander et al., 2015; Chadwick et al., 2019; Christiana et al., 2017; Edwards et al., 2011; Edwards et al., 2013; Edwards et al., 2014; Hennessy et al., 2010; Kristjansson et al., 2015; Nanney et al., 2008; Yousefian et al., 2009)
Transportation challenges	The need for car travel, lack of public transportation, absence of “late” buses; long distances between home and destinations	Barrier	*n* = 8 (Chadwick et al., 2019; Christiana et al., 2014; Edwards et al., 2011; Hennessy et al., 2010; Hinkle et al., 2018; Nanney et al., 2008; Palmer et al., 2024; Roberts, 2023; Tosa et al., 2018; Yousefian et al., 2009)
Safety concerns	Parental concern for injury or ‘stranger danger’ in outdoor spaces; social disorders and fear of crime	Barrier	*n* = 6 (Balvanz et al., 2016; Christiana et al., 2014; Flett et al., 2010; Hennessy et al., 2010; Meyer et al., 2019; Yousefian et al., 2009)
Cost/fees	Expensive fees for programs	Barrier	*n* = 5 (Alexander et al., 2015; Balvanz et al., 2016; Chadwick et al., 2019; Shah et al., 2019; Yousefian et al., 2009)
Economic challenges	Children from families of low socioeconomic status and schools with high free/reduced-price lunch enrollment had fewer opportunities	Barrier	*n* = 5 (Edwards et al., 2013; Galaviz et al., 2016; Kristjansson et al., 2015; Nanney et al., 2008; Yousefian et al., 2009)
Inadequate infrastructure	Lack of sidewalks; unpaved roads; heavy commercial traffic; unused open space; locked schoolyards	Barrier	*n* = 3 (Balvanz et al., 2016; Hennessy et al., 2010; Kristjansson et al., 2015)
Program design and implementation challenges	Delays in hiring staff; difficulty arranging transportation; misaligned priorities among staff; lack of community ownership; overly complicated activities within short timeframe; participation in online or technology-dependent programs	Barrier	(*n* = 3) (Palmer et al., 2024; Pate et al., 2003; Roberts, 2023)
Weather	Extreme weather conditions restricted school space and limited outdoor activity	Barrier	*n* = 3 (Chadwick et al., 2019; Hennessy et al., 2010; Sharaievska et al., 2019)
Limited staff	Limited human capital to sustain initiatives	Barrier	*n* = 2 (Chadwick et al., 2019; Edwards et al., 2014)
Community context and demographics	COVID-19 related barriers; prevalent drug use	Barrier	*n* = 2 (Kellstedt et al., 2022; Kristjansson et al., 2015)
Availability of programs and facilities	Presence of a variety of sports opportunities, community program, play areas, parks, walking trails, recreation centers	Facilitator	*n* = 11 (Alexander et al., 2015; Brett et al., 2002; Chadwick et al., 2019; Christiana et al., 2014; Edwards et al., 2011; Edwards et al., 2014; Kasehagen et al., 2012; Kristjansson et al., 2015; Leslie et al., 2016; Prochnow et al., 2020; Yousefian et al., 2009)
Resources and infrastructure	Access to physical activity equipment; provision of exercise clothes/gear; presence of natural resources; use of fitness trackers; technology that increased engagement	Facilitator	*n* = 7 (Chadwick et al., 2019; Christiana et al., 2014; Edwards et al., 2014; Hennessy et al., 2010; Palmer et al., 2024; Shah et al., 2019; Sharaievska et al., 2019)
Transportation and accessibility	Providing transportation options; offering modified/flexible access to facilities	Facilitator	*n* = 5 (Chadwick et al., 2019; Hennessy et al., 2010; Nanney et al., 2008; Roberts, 2023; Yousefian et al., 2009)
Community policy and support	Community awareness regarding physical activity; investments in public spaces; ability to pool resources	Facilitator	*n* = 5 (Alexander et al., 2015; Edwards et al., 2014; Kristjansson et al., 2015; Meyer et al., 2021; Yousefian et al., 2009)
Safe environments	Communities perceived as safe	Facilitator	*n* = 4 (Alexander et al., 2015; Galaviz et al., 2016; Meyer et al., 2019; Yousefian et al., 2009)
Program quality design	Providing services specifically tailored to youth; offering noncompetitive activities (especially for girls); competition that was not overly difficult	Facilitator	*n* = 3 (Chadwick et al., 2019; Christiana et al., 2017; Flett et al., 2010)

**Social Level**			
**Participation Factor**	**Description**	**Direction of Impact**	**Number of Studies (Citations)**
Lack of social support and cultural norms	Youth reported absence of friends in PA programs; presence of sedentary adults; normalization of fast food consumption and watching television; narrow conception of ‘exercise’	Barrier	*n* = 6 (Brett et al., 2002; Christiana et al., 2014; Flett et al., 2010; Meyer et al., 2019; Pate et al., 2003; Sharaievska et al., 2019)
Family responsibilities and scheduling	Parents’ busy work schedules; family responsibilities	Barrier	*n* = 3 (Brown et al., 2018; Chadwick et al., 2019; Yousefian et al., 2009)
Group dynamics and inclusivity	Existing, exclusive social networks within communities; discipline issues; racial divisions influenced perceptions of opportunities	Barrier	*n* = 2 (Edwards et al., 2014; Pate et al., 2003)
Family support and role models	Family members serving as role models, providing tangible support, and providing encouragement; parental modeling	Facilitator	*n* = 9 (Alexander et al., 2015; Brett et al., 2002; Christiana et al., 2014; Flett et al., 2010; Melton et al., 2018; Shah et al., 2019; Yousefian et al., 2009; Zuest, 2020)
Peer support	Relationships with friends; the presence of other active children; social connections; peer encouragement	Facilitator	*n* = 8 (Balvanz et al., 2016; Christiana et al., 2014; Flett et al., 2010; Palmer et al., 2024; Pate et al., 2003; Prochnow et al., 2020; Smith et al., 2022; Zuest, 2020)
Program staff	Experienced, diverse, and well-trained staff; co-participation (adult with child); clear program goals; supportive environments from program leaders	Facilitator	*n* = 3 (Meyer et al., 2019; Pate et al., 2003; Roberts, 2023)

**Individual Level**			
**Participation Factor**	**Description**	**Direction of Impact**	**Number of Studies (Citations)**
Lack of interest/motivation	Youth reported lack of internal drive for physical activity, preference for sedentary behaviors, and/or personal discomfort or disinterest	Barrier	*n* = 9 (Barfield et al., 2021; Brett et al., 2002; Brown et al., 2018; Christiana et al., 2014; Kristjansson et al., 2015; Palmer et al., 2024; Sharaievska et al., 2019; Thunfors et al., 2009; Yousefian et al., 2009)
Time constraints and prioritization	Youth reported conflicts with schoolwork, job commitments, social activities	Barrier	*n* = 3 (Chadwick et al., 2019; Palmer et al., 2024; Thunfors et al., 2009)
Low self-efficacy	Youth reported low self-efficacy for activities (e.g., outdoor recreation, weight lifting); Teachers reported low self-efficacy for delivering programs	Barrier	*n* = 2 (Roberts, 2023; Thunfors et al., 2009)
Previous negative experiences	Youth perceived some programs as too competitive; past negative experience or injuries during outdoor activities	Barrier	*n* = 2 (Christiana et al., 2014; Flett et al., 2010)
Motivation	Youth reported passion for sport; motivation; personal choice; desire to feel better; general interest in physical activity	Facilitator	*n* = 5 (Brett et al., 2002; Christiana et al., 2014; Flett et al., 2010; Palmer et al., 2024; Zuest, 2020)
Enjoyment	Youth reported appreciation for nature; fun/enjoyment; previous positive experience in outdoor participation;	Facilitator	*n* = 3 (Christiana et al., 2014; Flett et al., 2010; Palmer et al., 2024)
Competence and skill development	High perceptions of physical competence and skill building	Facilitator	*n* = 3 (Barfield et al., 2021; Palmer et al., 2024; Zuest, 2020)

Table 3 provides the quality assessment results using the AXIS Tool. Most studies (*n* = 35, 89.7%) were identified as being of high quality and low risk of bias. The remaining studies (*n* = 4) were identified as being of moderate quality and moderate risk of bias. Common methodological limitations in the moderate-quality studies included insufficient reporting of basic demographic data, limited information about non-responders or reasons for non-participation, and incomplete discussion of study limitations.

**Table 3:** Quality assessment scores using the appraisal tool for cross-sectional studies (AXIS) tool

Study ID	Q1	Q2	Q3	Q4	Q5	Q6	Q7	Q8	Q9	Q10	Q11	Q12	Q13	Q14	Q15	Q16	Q17	Q18	Q19	Q20	Overall Study Quality
Alexander et al., 2015 [28]	1	1	0	1	1	1	0	1	1	1	1	1	1	0	1	1	1	1	1	1	85%
Balvanz et al., 2016 [29]	1	1	0	1	1	0.5	0	1	1	1	1	1	0.5	0	1	1	1	1	0	1	75%
Barfield et al., 2021 [30]	1	1	0	1	1	1	0	1	1	0	1	1	0.5	0	1	1	1	1	1	1	78%
Brett et al., 2002 [31]	1	1	0	1	1	1	0	1	1	0.5	1	1	1	0	1	1	1	1	1	0.5	80%
Brown et al., 2018 [32]	1	1	0	1	1	0.5	0	1	1	1	1	1	1	1	1	1	1	1	1	1	88%
Chadwick et al., 2019 [33]	1	1	0	1	1	1	0	1	1	0.5	1	1	0.5	0	1	1	1	1	1	1	80%
Christiana et al., 2014 [34]	1	1	0	1	1	1	0	1	1	0.5	1	1	1	0	1	1	1	1	1	1	83%
Christiana et al., 2017 [35]	1	1	0	1	0.5	1	0.5	1	1	1	1	1	0.5	0	1	1	1	1	0.5	1	80%
Edwards et al., 2011 [36]	1	1	1	1	1	1	1	0.5	1	1	1	1	1	1	1	1	1	1	1	0.5	95%
Edwards et al., 2013 [37]	1	1	1	1	1	1	1	1	1	1	1	1	1	1	1	1	1	1	1	1	100%
Edwards et al., 2014 [38]	1	1	1	1	1	1	0	1	1	0.5	1	1	1	0.5	1	1	1	1	1	1	90%
Flett et al., 2010 [39]	1	1	1	1	0.5	0.5	1	1	1	1	1	1	1	1	1	1	1	1	0.5	1	93%
Galaviz et al., 2016 [40]	1	1	1	1	1	1	1	1	0.5	1	1	1	1	1	1	1	1	1	1	0.5	95%
Gay et al., 2011 [41]	1	1	0	1	0.5	0.5	0.5	1	1	1	1	1	1	1	1	1	1	1	1	1	88%
Hennessy et al., 2010 [42]	1	1	0	1	0.5	1	0.5	1	1	1	1	1	1	1	1	1	1	1	1	1	90%
Hinkle et al., 2018 [43]	1	1	0	1	0.5	0.5	0.5	1	0.5	1	0	1	1	0	1	1	1	0	1	0.5	68%
Kasehagen et al., 2012 [44]	1	1	1	1	1	1	1	1	1	1	1	1	1	1	1	1	1	1	0.5	0.5	95%
Kellstedt et al., 2021 [45]	1	1	0	0	0.5	0.5	0.5	1	1	0	0.5	1	1	0	1	1	1	1	0	1	65%
Kellstedt et al., 2022 [46]	1	1	0	1	1	1	0.5	1	1	1	1	1	1	0	1	1	1	1	0	1	83%
Kristjansson et al., 2015 [47]	1	1	0	1	1	1	0	1	1	1	1	0	1	1	0	1	1	1	0	1	75%
Leslie et al., 2016 [48]	1	1	1	1	0.5	1	0	1	0	1	1	1	1	1	1	1	1	1	0.5	1	85%
MacDowell et al., 2011 [49]	0	0	0	1	1	1	0	1	1	1	1	1	1	0	1	1	1	0	1	0.5	68%
McBride et al., 2017 [50]	1	0.5	0	1	1	0.5	0.5	1	1	1	1	1	1	0	1	1	1	1	0.5	0.5	78%
Melton et al., 2018 [51]	1	1	0	1	0.5	0.5	0	1	1	1	0	1	0	0	1	1	1	1	1	0.5	68%
Meyer et al., 2019 [52]	1	1	1	1	1	1	0.5	1	1	1	1	1	1	0.5	1	1	1	1	1	1	95%
Meyer et al., 2021 [53]	1	1	0	1	1	1	0.5	1	0.5	0.5	1	1	1	0.5	1	1	1	1	0.5	1	83%
Molitor & Naber, 2024 [54]	1	1	0	1	1	0.5	0.5	1	1	1	1	1	1	0	1	1	1	1	1	1	85%
Nanney et al., 2008 [55]	1	1	0	1	1	1	1	1	1	1	1	1	1	1	1	1	1	1	0	0.5	88%
Palmer et al., 2024 [56]	1	1	0	1	1	0.5	0	1	1	0.5	1	1	0.5	0	1	1	1	1	0.5	1	75%
Pate et al., 2003 [57]	1	1	0	1	0.5	1	1	1	1	1	1	1	1	1	1	1	1	1	1	1	93%
Prochnow et al., 2020 [58]	1	1	1	1	1	1	0.5	1	1	1	1	1	1	0.5	1	1	1	1	1	1	95%
Roberts 2023 [59]	1	1	0	1	1	1	0.5	1	1	1	0	1	1	1	1	1	1	1	1	1	88%
Shah et al., 2019 [60]	0	1	1	1	1	1	0.5	1	1	1	1	1	1	1	1	1	1	1	0.5	1	90%
Sharaievska et al., 2019 [61]	1	1	1	1	0.5	0.5	0.5	1	1	1	1	1	1	1	1	1	1	1	1	1	93%
Smith et al., 2022 [62]	1	1	1	1	1	1	0.5	1	1	1	1	1	1	1	1	1	1	1	1	1	98%
Thunfors et al., 2009 [63]	1	1	1	1	1	1	0.5	1	1	1	1	1	1	1	1	1	1	1	1	1	98%
Tosa et al., 2018 [64]	1	1	0	1	1	1	0	1	1	0	1	0	0.5	0	1	1	1	1	1	1	73%
Yousefian et al., 2009 [65]	1	1	0	1	1	1	0.5	1	1	1	0	1	1	1	1	1	1	0	1	0.5	80%
Zuest 2020 [66]	1	1	0	1	1	1	0.5	1	1	1	1	1	1	0	1	1	1	1	1	1	88%

Point System: 1 for Yes, 0 for No, 0.5 for Don’t Know (Q13 and Q19 were reverse coded)

≥ 70% = High Quality/Low Risk of Bias; 50–69% = Moderate Quality/Moderate Risk of Bias; < 50% = Low Quality/High Risk of Bias

### Barriers and facilitators

Barriers and facilitators to out-of-school, structured PA participation are presented at the program/environmental, social, and individual levels. Over 71% (*n* = 28) studies reported at least one program/environmental factor, 49% (*n* = 19) of studies reported at least one social participation factor, and about 33% (*n* = 13) reported at least one individual level factor of participation.

### Environment and program level barriers

The most frequently reported barrier to structured, out-of-school PA participation among rural youth was the lack of facilities and programs (*n* = 10 studies). Studies consistently highlighted limited numbers of venues, health programs, indoor and outdoor facilities, and extracurricular activities available to rural youth [28, 33, 35–38, 42, 47, 55, 65]. Several studies specifically noted the lack of sports opportunities available for girls, reflecting gender-specific programmatic gaps in rural communities [28, 35–37]. 

Transportation emerged as another significant barrier, identified across eight studies. Rural youth and their families faced challenges related to the need for car travel, lack of public transportation systems, and/or absence of late buses that could facilitate participation in after-school programs [33, 34, 36, 42, 55, 56, 64, 65]. The long distances between homes and activity destinations in rural areas compounded these transportation difficulties, creating substantial logistical barriers for families seeking to enroll their children in structured PA programs [34, 36, 42]. 

Financial barriers represented a substantial obstacle to participation, manifesting in two distinct ways across the reviewed studies. Five studies identified direct cost and fee barriers, with families reporting that expensive program fees prevented participation [28, 29, 33, 60, 65]. Additionally, five studies highlighted broader economic challenges, noting that children from families of low socioeconomic status and schools with high free and reduced-price lunch enrollment had fewer opportunities for structured PA participation [37, 40, 47, 55, 65]. 

Infrastructure and environmental barriers also posed significant challenges for rural youth. Three studies documented inadequate infrastructure, including lack of sidewalks, unpaved roads, heavy commercial traffic, unused open spaces, and locked schoolyards that limited access to PA opportunities [29, 42, 47]. Safety concerns were reported in six studies, encompassing both parental concerns about injury or “stranger danger” in outdoor spaces, as well as broader community issues such as social disorders and fear of crime that deterred participation [29, 34, 39, 42, 52, 65]. 

Operational and contextual barriers further complicated program delivery and participation. Three studies identified program design and implementation challenges, including delays in hiring staff, difficulty arranging transportation, misaligned priorities among staff, lack of community ownership, overly complicated activities within short timeframes, and challenges with online or technology-dependent programs [56, 57, 59]. Limited staffing capacity was noted in two studies as a barrier to sustaining PA initiatives [33, 38]. Environmental factors, particularly extreme weather conditions that restricted school space and limited outdoor activities, were reported as barriers in three studies [33, 42, 61]. Finally, two studies highlighted community context and demographic factors, including COVID-19 related barriers (e.g., increased demands on parents) and prevalent drug use, that negatively impacted youth participation in structured PA programs [46, 47]. 

### Environment and program level facilitators

The most frequently reported facilitator to structured, out-of-school PA participation among rural youth was the availability of programs and facilities (*n* = 11 studies). Studies consistently emphasized the importance of having a variety of sports opportunities, community programs, play areas, parks, walking trails, and/or recreation centers accessible to rural youth [28, 31, 33, 34, 36, 38, 44, 47, 48, 58, 65]. The presence of diverse PA options was particularly important in rural settings where programmatic choices were often limited. Accessibility emerged as a critical facilitator across five studies, with researchers highlighting the importance of providing transportation options and/or offering modified or flexible access to facilities [33, 42, 55, 59, 65]. These accommodations were essential for overcoming the geographic and logistical challenges inherent in rural communities, enabling families to participate despite transportation barriers and scheduling constraints.

Community-level support and policy initiatives served as important facilitators in five studies. Key elements included community awareness regarding PA, investments in public spaces, and/or the ability to pool resources across organizations and stakeholders [28, 38, 47, 53, 65]. This collective approach was particularly valuable in rural settings where individual organizations might lack sufficient resources to sustain programs independently. Environmental safety represented another significant facilitator, with four studies identifying communities perceived as safe as crucial for youth PA participation [28, 40, 52, 65]. When parents and youth felt secure about community safety, participation rates in outdoor and community-based activities increased substantially. Program quality and design elements emerged as facilitators in three studies, emphasizing the importance of providing services specifically tailored to youth needs [33, 35, 39]. Key design features included offering noncompetitive activities, particularly for girls, and ensuring that competitive elements were not overly difficult or intimidating for participants. These tailored approaches helped address the diverse interests and skill levels present among rural youth populations.

Finally, seven studies identified resources and infrastructure as important facilitators to participation. These included access to PA equipment, provision of exercise clothes and gear, presence of natural resources, use of fitness trackers, and/or technology that increased engagement [33, 34, 38, 42, 56, 60, 61]. The provision of necessary equipment and gear was particularly important for rural families who might face financial constraints in purchasing such items independently.

### Social barriers

The absence of social support and prevailing cultural norms emerged as significant social barriers to PA participation, identified across six studies. Youth frequently reported the absence of friends in PA programs, which reduced their motivation to participate [31, 34, 39, 52, 57, 61]. Additionally, studies documented the presence of sedentary adults in rural communities who modeled inactive behaviors, along with the normalization of fast food consumption and television watching as primary leisure activities [31, 52]. These cultural patterns contributed to narrow conceptions of “exercise” that limited youth engagement in diverse forms of PA. Family responsibilities and scheduling conflicts represented another category of social barriers, reported in three studies. Parents’ busy work schedules and competing family responsibilities often prevented consistent participation in structured PA programs [32, 33, 65]. These scheduling challenges were particularly pronounced in rural communities where parents might work multiple jobs or have extensive commutes, leaving limited time for transporting children to activities or providing supervision. Group dynamics and inclusivity issues created additional social barriers in two studies. Researchers identified existing, exclusive social networks within rural communities that could make it difficult for newcomers or certain groups to feel welcome in PA programs [38, 57]. Discipline issues and racial divisions further influenced perceptions of opportunities, with some youth feeling excluded or unwelcome in certain programs or facilities based on social hierarchies or discriminatory attitudes present in their communities.

### Social facilitators

Family support and positive role modeling emerged as the most frequently reported social facilitator, identified across nine studies. Family members who served as role models, provided tangible support, and/or offered encouragement significantly enhanced youth participation in PA programs [28, 31, 34, 39, 51, 60, 65, 66]. Parental modeling of active behaviors was particularly influential, demonstrating to youth that PA was valued and prioritized within the family unit [31, 60, 66]. Peer support represented another crucial social facilitator, documented in eight studies [28, 31, 34, 39, 51, 60, 65, 66]. Strong relationships with friends, the presence of other active children, social connections, and/or peer encouragement all contributed to increased participation rates [29, 34, 39, 56–58, 62, 66]. The social aspect of PA was particularly important for rural youth, who might have fewer opportunities for peer interaction compared to their urban counterparts. Program staff characteristics and approaches served as important social facilitators in three studies. Experienced, diverse, and well-trained staff members who could relate to rural youth enhanced program appeal and effectiveness [52, 57, 59]. Additional staff-related facilitators included co-participation opportunities where adults engaged in activities alongside children, clear program goals that provided structure and direction, and supportive environments created by program leaders who understood the unique needs and challenges of rural youth populations.

### Individual level barriers

Lack of interest and motivation represented the most frequently reported individual barrier to PA participation, identified across nine studies. Youth consistently reported a lack of internal drive for PA, preference for sedentary behaviors, and/or personal discomfort or disinterest in available programs [30–32, 34, 47, 56, 61, 63, 65]. This lack of intrinsic motivation was particularly challenging in rural settings where program options were already limited, making it difficult to find activities that might spark youth interest. Low self-efficacy emerged as another individual barrier in two studies, manifesting in multiple ways within rural communities. Youth reported low self-efficacy for specific activities, such as outdoor recreation and weight lifting, while teachers and program staff reported low self-efficacy for delivering PA programs effectively [59, 63]. These confidence-related barriers created cycles where both youth and adults felt unprepared or unable to engage successfully in PA programming. Time constraints and prioritization challenges represented additional individual barriers, documented in three studies. Rural youth reported conflicts with schoolwork, job commitments, and social activities that competed with structured PA participation [33, 56, 63]. These scheduling conflicts were often more pronounced in rural areas where youth might have additional responsibilities such as farm work or longer commutes to school and activities. Previous negative experiences served as individual barriers in two studies, where youth perceived some programs as too competitive, or had past negative experiences or injuries during outdoor activities [34, 39]. These experiences created lasting impressions that deterred future participation, particularly in communities with limited program alternatives.

### Individual level facilitators

Motivation emerged as the primary individual facilitator, reported across five studies[31, 34, 39, 56, 66]. Youth who demonstrated passion for sport, general interest in PA, personal motivation, or general interest in PA were more likely to participate in structured programs [31, 34, 39, 56, 66]. Enjoyment was another individual level facilitator[34, 39, 56], and previous positive experiences in outdoor participation were particularly influential in sustaining long-term engagement, as youth who had enjoyed activities wanted to seek similar opportunities [39]. Competence and skill development represented another important individual facilitator, identified in three studies [30, 56, 66]. Youth with high perceptions of physical competence and those who recognized skill-building opportunities within programs showed increased participation rates [30, 56, 66]. 

## Discussion

The purpose of this study was to identify, describe, and synthesize peer-review literature on barriers and facilitators to participation in structured, out-of-school PA at multiple ecological levels for US youth living in rural areas. There were 39 studies that met the inclusion criteria. Participation factors were most commonly reported at the environmental/program level, followed by the social and individual levels. As analyzed by the AXIS Tool, the selected studies’ quality assessments showed that all but four of the articles could be regarded as high quality.

### Environment and program level

At the environment and program level, the most commonly reported participation factors were related to the availability of programs and facilities for structured PA programs [28, 31, 33–38, 42, 44, 47, 48, 55, 58, 65]. Supportive factors included having choices (e.g., variety of sport opportunities), local programming (e.g., community programs and recreation centers), and/or physical spaces for activities (e.g., parks, play areas, trails) [28, 31, 33–38, 42, 44, 47, 48, 55, 58, 65]. Conversely, the lack of these supports was a commonly reported barrier [28, 33, 35–38, 42, 47, 55, 65]. There was also evidence showing gender-based disparities, with girls being differentially affected by the lack of sports available to them [35]. Beyond the reported participation factors, it is important to consider the systematic economic and geographic constraints that contribute to this environment. Sparse populations across vast distances can hinder public investment in recreation infrastructure due to a smaller tax base and limiting the recruitment of specialized staff [67, 68]. This often forces programs to rely on temporary or multi-use space (e.g., school gyms)[69], which may compromise program consistency and quality. Further, limited population density in rural areas often prevents programs from reaching financially sustainable enrollment numbers [17]. 

The importance of PA programs and facilities in supporting youth program engagement and PA levels aligns with the larger literature base. A study conducted by Guerra et al. (2024) used data from a national survey of US adolescents and showed a significant association between the availability of school recreational facilities with youth MVPA [70]. Another study conducted among 928 adolescents by McCormack et al. (2023) showed that parental perceptions of multiple available recreation environments was positively associated with adolescents’ participation in teams and PA classes [71]. 

Resources, infrastructure, and accessibility were also commonly reported participation factors at the environment and program levels, particularly regarding transportation [29, 33, 34, 36, 42, 43, 47, 55, 56, 59, 64, 65]. This aligns with previous literature conducted both within and outside of rural communities, which identifies transportation as one of the primary determinants of PA participation [15, 65, 72]. Key factors identified in the present study included infrastructure around transportation to and from opportunities (e.g., sidewalks and roads), provision of transportation options, flexible facility access, lack of public transportation and/or ‘late buses’, and long travel distances [33, 42, 55, 59, 65]. Additionally, resources that enhanced program participation (e.g., PA equipment, technology) were identified as important facilitators at the program level [33, 34, 38, 42, 56, 60, 61]. It is posited that transportation and resource barriers are further exacerbated in rural areas given the longer distances between destinations and the often limited resources in these communities [9]. Notably, there have been calls to improve infrastructure in rural areas to support active living in these communities [9]. However, addressing environmental- and program-level participation barriers calls for strategies that address the unique needs and assets available in rural communities, with special consideration for resource-strained communities. Strategies could include shared use agreements between community organizations, schools, and the city to allow for increased access to facilities for PA. This approach is generally low-cost and has been shown to be effective in increasing PA engagement [73]. 

### Social level

At the social level, social support from family and peers was the most identified participation factor for structured, out-of-school PA. This included modeling of PA by parents, staff and peers; tangible and emotional support from parents; and/or positive peer connections (or lack thereof) [28, 39, 51, 56, 60, 66]. Social support from peers and parents are well-established as facilitators of PA participation among youth [74–77]. Although peer support is a consistent predicator of PA across age groups, peer support typically becomes more influential than parental support as youth age into adolescents [78]. Additionally, there is evidence to suggest that girls may receive less encouragement and tangible support for organized PA (e.g., sports) than boys [79, 80]. Thus, strategies aimed to increase social support should be tailored to youth needs, and should aim to develop a strong social network supportive of PA participation. For example, programs can include a home element (e.g., activities that can be done with family) or provide ‘family days’, in which families are invited to be active together as part of the out-of-school program [81]. Program staff support can be promoted through evidence-based training that addresses modeling and positive ‘team-building’ strategies so that the program curriculum can be designed to support peer support around PA[82, 83].

Although there is limited research examining social support needs by urbanicity or rurality, it is likely that these needs are informed by the physical environment[77, 84], which creates unique challenges within more rural communities. For example, rural youth may require tangible support from parents or the community to overcome logistical barriers such as transportation to PA opportunities [85]. Still, rural communities are often characterized by strong social networks and social capital[86], which provide invaluable opportunities to support youth engagement in organized PA. Building on these existing social networks can help create a positive community culture around keeping youth active.

### Individual level

Motivation, interest, and enjoyment among youth were the most commonly identified participation factors at the individual level. This included an internal drive or interest for PA (or lack thereof), previous positive experiences, a preference for sedentary behaviors (e.g., screen time), and/or personal discomfort or disinterest with the activities [30–32, 34, 39, 47, 56, 61, 63, 65, 66]. In the larger literature base, motivation, fun, or interest are among the most influential PA participation factors among youth [87–89]. Youth motivation/interest has also been shown to be driven by factors such as confidence or social support[90–93], which were also reported as participation factors within the presented studies [28–31, 34, 39, 51, 52, 56–63, 65, 66]. Motivation, fun, and interest needs have been shown to vary among youth populations based on factors such as age and gender[94–96], and although there is limited evidence around how these factors may differ by urbanicity and rurality, some studies suggest there may be rural-urban differences in youth preferences for outdoor activities and fitness promotion technologies [30, 97]. Collectively, individual level factors within the present study included elements such as motivation, competence, and skill-building – components that are integral to physical literacy, in which individuals have the confidence, physical competence, motivation, and knowledge to be physical active for life [98]. This further emphasizes the importance of and need for quality PA programming for youth. Out-of-school PA programs in rural programs should incorporate the interests of local youth populations while promoting positive peer connections and building confidence around physical activities. Programs should also consider how these needs may vary across gender, ages, abilities, and past experiences with physical activities or programs. Program design and tailoring can include collaborative co-design through youth advisory boards and community listening sessions[99], which can help leverage social capital and promote youth voices within the design process.

Table 4 provides a summary of recommendations for addressing participation in out-of-school, structured PA among rural youth. Strategies to increase participation should consider how programs can identify and address individual- (e.g., fun, engaging, activities) and social-level (e.g., promoting peer and family support for program support) participation factors within the context of the program and environment factors (e.g., unique facilitators/barriers within rural communities - resource and funding restraints, access to outdoor spaces, community networks). Although this review contributes to a gap in the literature, additional research is needed to examine participation factors for out-of-school, structured PA among rural youth so that these programs can be designed and adapted to meet the unique needs of this population.

**Table 4:** Summary table of recommendations

Recommendations for Addressing Participation in Structured, Out-of-school Physical Activity among Rural Youth in the United States
• Program Design and Infrastructure
o Provide diverse physical activity options accommodating varied interests and skill levels
o Ensure consistent access to adequate physical spaces for programming
o Secure essential resources (equipment, technology, materials) supporting quality programming
• Transportation and Access
o Establish infrastructure enabling safe active transportation (walking/biking routes)
o Implement flexible facility scheduling to minimize transportation barriers
o Provide transportation solutions (extended bus services, carpooling programs)
o Advocate for accessible public transportation serving program locations
• Collaborative Resource Sharing
o Establish formal shared-use agreements among community organizations, schools, and local government to maximize facility access
• Social Support
o Integrate family-engagement components enabling home-based participation
o Host family activity events fostering intergenerational physical activity
o Provide evidence-based professional development emphasizing positive role modeling and team-building strategies
o Offer tangible support addressing logistical barriers (equipment, fee assistance)
o Leverage existing community social networks and relationships
• Youth-Centered Approach
o Employ participatory design methods (youth advisory boards, community listening sessions)
o Tailor programming to developmental needs, gender, ability levels, and prior experiences
o Build physical competence alongside enjoyment to enhance self-efficacy and sustained engagement

It is of note that nearly 70% of the included studies were either missing a definition of rural or used a generic definition of rurality. While this makes it difficult to determine community characteristics that are important for PA within these studies, this challenge persists across rural health-related literature. Future publications need to include a description of how rurality was determined to allow readers to better assess how the context of communities included in studies compare to other rural areas. Common classification systems include the RUCA (Rural-Urban Commuting Area) codes (based on population density and primary commuting flows to surrounding areas)[100], RUCC (Rural-Urban Continuum Codes; based on urbanization and adjacency to a metro area)[101], and the US Census Bureau’s definition (i.e., all geographic areas that are not classified as urban) [102]. 

### Limitations

Limitations of this study should be noted. As is the case with all systematic literature reviews, it is possible that an article meeting inclusion criteria was not identified through our search process. However, we attempted to mitigate this risk by using multiple reviewers and multiple databases, as well as the Covidence academic search software. Comparability across studies may be limited by variability in study design and measures. This review was limited to English-language studies conducted in the US, which may limit the generalizability of findings to other countries with rural populations. Future research examining participation factors across international contexts would provide valuable comparative insights.

## Conclusions

This systematic review of the literature identified studies that examined participation factors for structured, out-of-school PA programs at multiple ecological levels among US rural youth. Additional research is needed on barriers and facilitators of youth engagement in these programs. Participation factors were identified at multiple ecological levels (e.g., environmental/program, social, individual) and included factors such as facilities and programs, transportation, resources and infrastructure, social support, and interest/motivation. Factors at the social and individual levels were consistent with the larger evidence base, but efforts to address related barriers should account for the environmental/program-level contexts that are often unique for rural settings. These findings address a gap in the literature and can be used to shape policy, improve out-of-school program design, and inform targeted strategies to address gender and geographic disparities in youth activity levels.

## Supplementary Information

Supplementary Material 1.

## Data Availability

Data sharing is not applicable to this article as no new datasets were generated during the current study.

## Abbreviations

AXIS: Appraisal tool for cross-sectional studies
CSPAP: Comprehensive School Physical Activity Program
PA: Physical activity
US: United States
