# Supplementary material for: Barriers and facilitators to structured, out-of-school physical activity participation among rural youth in the United States: a systematic review
Source: BMC Public Health. 2026 Jan 31;26:754. doi: 10.1186/s12889-026-26205-x (PMC12947335; doi:10.1186/s12889-026-26205-x)
Supplement: Supplementary file 1 — Supplementary Material 1. [file 12889_2026_26205_MOESM1_ESM.docx]

**Supplementary File 1.** Detailed Search Strategy

((out-of-school program OR after-school program OR before-school program OR extracurricular activit* OR afterschool activit* OR out-of-school time OR extended day program OR extra-curricular program OR youth development program* OR summer program OR holiday program OR co-curricular activit* OR latchkey program OR youth club OR organized activit* OR structured activit* OR youth organization OR sport program OR youth empowerment program OR leadership program OR service learning program OR YMCA OR Boys & Girls Club OR 4-H program OR Scout program OR community program OR youth program OR camp OR recreation) AND (physical activit* OR exercis* OR physical fitness OR physical education OR sport OR workout OR active play OR active transport* OR recreation) AND (youth OR young people OR adolescen* OR teen OR teenager OR juvenile OR minor OR child* OR young adult OR tween OR pre-teen OR preteen OR school-age OR school age OR student OR pupil) AND ( rural* OR town OR village OR remote area OR non-metropolitan OR non-urban))

**Supplementary File 2.** Extraction Template

1. Study Title
2. Lead Author Last Name
3. Study Publication Year
4. Study Type
   1. Quantitative
      1. Longitudinal
      2. Cross-sectional
   2. Qualitative
   3. Mixed Methods
   4. Other
5. Setting/Cohort if applicable
6. Location (State(s), Cities)
7. Time Period of Data Collection (Month and Year)
8. Definition of rural
   1. Generic urban/rural classification
   2. Census tracts
   3. ZIP code tabulation
   4. County-level
   5. OMB definitions of metro and micropolitan statistical areas
   6. USDA-ERS
   7. RUCA
   8. RCC
   9. UIC
   10. FAR
   11. Federal Office of Rural Health Policy at the HRSA
   12. NCHS at CDC definition
   13. CBSAs
   14. Department of Education definition
   15. Other
9. Participant Characteristics
   1. Type (e.g., youth, parent, staff)
   2. Sample Size
   3. Mean Age
   4. Percent Male/Female
   5. Race/ethnicity breakdown
10. Out of School Structured Activities Included
11. Descriptive Statistics on Participation in out of school structured activities
    1. Rate of participation
    2. Statistical differences explored between boys/girls
    3. Statistical differences explored by other child demographics
12. Barriers to Participation
13. Facilitators of Participation
14. Methods used to assess barriers/facilitators
    1. Survey Type
    2. Interviews (semi-structured, focus group, etc.)
    3. Parent vs. self-report
15. Funding sources
16. Reported conflicts of interest
17. Study Quality – AXIS Tool
